# Gray Matter Abnormalities in Myotonic Dystrophy Type 1: A Voxel-Wise Meta-Analysis

**DOI:** 10.3389/fneur.2022.891789

**Published:** 2022-07-07

**Authors:** Qirui Jiang, Junyu Lin, Chunyu Li, Yanbing Hou, Huifang Shang

**Affiliations:** Department of Neurology, Laboratory of Neurodegenerative Disorders, West China Hospital, Sichuan University, Chengdu, China

**Keywords:** myotonic dystrophy type 1, voxel-based morphometry, gray matter volume, meta-analysis, neuroimag

## Abstract

**Background:**

A growing number of voxel-based morphometry (VBM) studies have demonstrated widespread gray matter (GM) abnormalities in myotonic dystrophy type 1 (DM1), but the findings are heterogeneous. This study integrated previous VBM studies to identify consistent GM changes in the brains of patients with DM1.

**Methods:**

Systematic retrieval was conducted in Web of Science, Pubmed, and Embase databases to identify VBM studies that met the inclusion requirements. Data were extracted. The Seed-based d Mapping with Permutation of Subject Images (SDM-PSI) software was used for meta-analysis of voxel aspects.

**Results:**

A total of eight VBM studies were included, including 176 patients with DM1 and 198 healthy controls (HCs). GM volume in patients with DM1 was extensively reduced compared with HCs, including bilateral rolandic operculum, bilateral posterior central gyrus, bilateral precentral gyrus, right insula, right heschl gyrus, right superior temporal gyrus, bilateral supplementary motor area, bilateral middle cingulate gyrus/paracingulate gyrus, left paracentral lobule, and bilateral caudate nucleus. Meta-regression analysis found that regional GM abnormalities were associated with disease duration and Rey-Osterrieth Complex Figure (ROCF)-recall scores.

**Conclusion:**

DM1 is not only a disease of muscle injury but also a multisystem disease involving brain motor and neuropsychiatric regions, providing a basis for the pathophysiological mechanism of DM1.

## Introduction

Myotonic dystrophy type 1 (DM1) is an autosomal dominant genetic disorder caused by CTG's abnormal repeat expansion in the noncoding region of the dystrophia myotonic protein kinase (*DMPK*) gene. It is the most common muscular dystrophy in adults with a global prevalence between 1/3,000 and 1/8,000 (1). It is a multisystem disease that affects not only the musculoskeletal system but also the respiratory, gastrointestinal, genitourinary, and central nervous system. The main clinical manifestations of DM1 include myotonia, muscle weakness, mild intellectual disability, speech delay, fatigue, impaired attention and memory, and excessive daytime sleepiness (2), resulting in a substantial disease burden and impairment across many different domains of patients' lives (3). There are no disease-modifying treatments for the disease currently. Knowing about the neuroimaging changes and the associations between affected areas in the whole brain and clinical manifestations such as muscular dystrophy and cognitive impairment can help us to better understand the pathogenesis and explore new therapeutic targets.

Over the years, various morphologic MRI techniques have been used to reveal brain structural changes in DM1, mainly including conventional morphological brain MRI, voxel-based morphometry (VBM), and diffusion tensor imaging (DTI). VBM can quantitatively analyze the changes in density or volume of gray matter (GM) and white matter (WM) of each voxel in MRI, thus reflecting the differences in corresponding anatomical structures. It is a new method of evaluating GM and WM lesions in the whole brain. In the past, many studies based on VBM explored GM changes in patients with DM1 and their correlation with clinical features. Numerous studies have shown cortical atrophy in patients with DM1, including the frontal, parietal, cingulate gyrus, and temporal lobes (4–10). Some studies showed atrophy of the striatum (4, 6, 7, 9–13), while others did not (5, 8, 14). Some studies revealed cerebellar GM atrophy in patients with DM1 (5, 8, 12). However, Labayru et al. found that compared to healthy controls (HCs), patients with DM1 had increased cerebellar GM volume at baseline and decreased cerebellar volume after approximately 9 years of follow-up (10). Therefore, the results were inconsistent, probably due to varied sample sizes, the characteristics of patients, disease duration, and methods.

At present, there is no meta-analysis to quantitatively analyze the changes in GM volume (GMV) in patients with DM1 compared with HCs. Hence, it is of great significance to select appropriate methods for quantitative analysis. Seed-based d Mapping (SDM) is a statistical technique for meta-analyses studies on differences in brain activity or structure in which neuroimaging technique such as fMRI, VBM, DTI, or PET is used. The methods have been fully validated in many studies (15, 16). SDM with Permutation of Subject Images (SDM-PSI) is a new algorithm that can use standard voxel-wise tests and conducts a standard PSI. SDM-PSI version 6.21 is the latest version, it includes the following new features: (nearly) unbiased estimation of effect sizes and familywise correction for multiple comparisons (15). In this study, our aim was to conduct a voxel analysis of GMV changes in patients with DM1 using the SDM-PSI method, to clarify the GMV changes in patients and their correlation with clinical manifestations.

## Methods

### Search and Inclusion of Studies

We conducted a systematic and detailed search of works in Pubmed, Embase, and Web of Science databases from their inception to October 17, 2021. The following keywords are used to search in these databases: (“myotonic muscular dystrophy” OR “myotonic dystrophy” OR “dystrophy Myotomic”) AND (“VBM” OR “voxel-based morphometry” OR “gray matter” OR “white matter”). Additional searches were also conducted in the reference list and related articles. We included studies in our meta-analysis when the article met the following criteria: (1) was published as an original article in a journal and peer-reviewed; (2) the enrolled patients were clearly diagnosed with DM1; (3) reported changes in GMV in patients with DM1 compared to HC, with three-dimensional coordinates (*x, y*, and *z*) based on standard stereo space (i.e., Talairach or Montreal Neurological Institute space); and (4) reported significance thresholds that were corrected for multiple comparisons or uncorrected with spatial extent thresholds. Studies were excluded if (1) there were no HCs or definite diagnosis of DM1 in the article; (2) only the region of interest in the brain rather than the whole brain was analyzed; and (3) the article lacked a spatial coordinate value or significance test. For patients from the same institution, we chose the study with a larger sample size.

### Data Extraction and Quality Assessment

Extract the following data for each article: numbers of patients and controls, mean age, mean disease duration, the mean number of CTG repeats, muscular impairment rating scale (MIRS) scores, beck depression inventory (BDI) scores, technical parameters of image scanning, structural data analysis software, and other clinical data. In addition, according to the SDM tutorial the peak coordinates and their effect sizes (*t* values, *z* scores, or *p* values) of abnormal GM areas in each article were extracted. Two authors (Qirui Jiang and Junyu Lin) reviewed the studies independently. A 15-point checklist (Supplementary Table 1) was used for each study quality assessment, which was described in previous meta-analyses (17, 18). Each item is rated 2, 1, or 0 depending on how well the criteria are met. This checklist provides a reference for the rigor of the included study.

### Meta-Analysis of Studies

We used SDM-PSI version 6.21 to perform an intelligent voxel meta-analysis of GMV differences in brain regions between patients with DM1 and HCs. The meta-analysis of VBM studies is described in detail in the software tutorial (www.sdmproject.com) and previous studies (16, 19). In this study, we first established a file containing sample size, clinical data, and statistical thresholds. In addition, the spatial coordinates of GMV differences between DM1 and HCs in each article and corresponding *t* statistics were extracted according to the required format. If there were no statistics, we use software to convert *z* scores or *p* values into *t* statistics, and input the obtained data into the software according to the format requirements. The software will use maximum likelihood techniques to estimate the most likely effect size and its standard error, and a random-effects model will be used for the meta-analysis of each study. Then we conducted family-wise error correction for multiple comparisons and establish statistical significance. We used the following parameters in the software: anisotropy=1, full width at half maximum = 20 mm, voxel size = 2 mm, imputations = 50, permutations = 1,000, *p* = 0.005, peak height threshold = 1, extent threshold = 10 voxels, and nonthreshold cluster enhancement family-wise error rate *p* = 0.05. It has been proved that these parameters can balance false negative and false positive well and have high sensitivity and specificity.

*Q* and *I*^2^ statistics were calculated to assess heterogeneity between included studies. *I*^2^ > 50% was considered to be highly heterogeneous. Egger's test and funnel plot were used to detect publication bias. *p* < 0.05 or an asymmetry plot were considered significant. Finally, jackknife sensitivity was performed, that is, the analysis was repeated after each study was deleted to see whether the results were changed. Meta-regression analysis was performed on clinical characteristics such as age, disease duration, MIRS, and BDI, with *p* = 0.005 and extent threshold = 10 voxels.

## Results

### Included Studies

**Figure 1:**
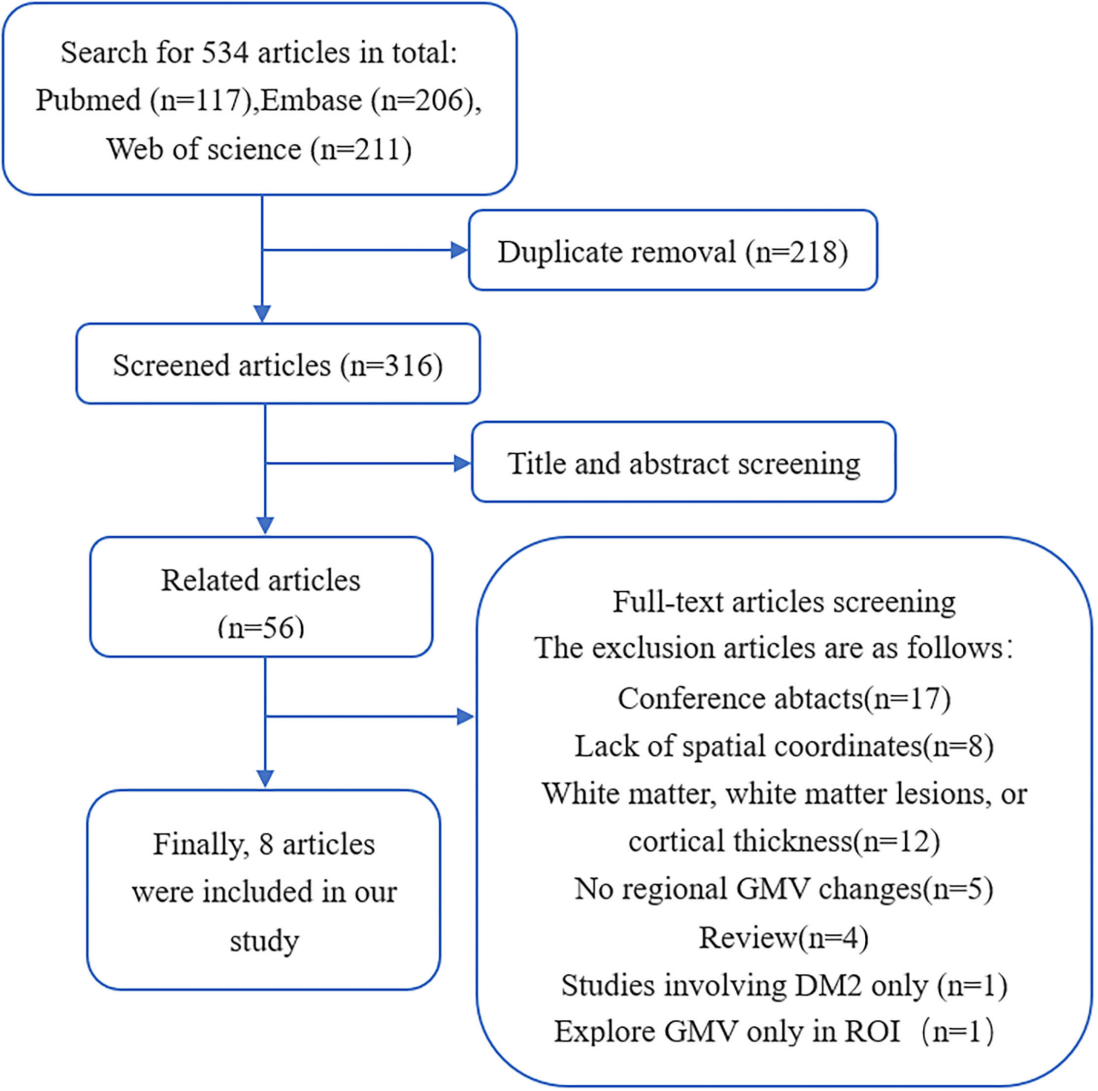
The screening process of articles.

After searching systematically, 316 potentially related studies were initially found, and finally, 8 studies met the inclusion criteria and were analyzed in the meta-analysis (4–11). The detailed process of inclusion and exclusion is shown in Figure 1. Because one of the studies divided patients into two groups, namely, pediatric onset and adult onset, and respectively contrasted with HCs, it was included as two separate studies (10). The 15-point checklist was used to score the quality of each article, and the results of each article were all greater than or equal to 13 points. Ultimately, our study included 176 patients with DM1 and 198 HCs. The demographic, clinical characteristics, and quality assessment of the included studies are summarized in Table 1.

**Table 1:** Demographic, clinical characteristics, and quality assessment of the included 8 studies.

**Study**	**Sample size**	**Age (years)**	**Sex (F/M)**	**Disease duration (years)**	**CTG repeats**	**MIRS**	**MMSE**	**TMT**	**ROCF**	**BDI**	**Software, scanner (T)**	**Threshold**	**Quantity score**
Zanigni et al. (9)	DM1 24 HC 25	38.5 ± 11.8 38.5 ± 11.3	10/14 11/25	16.2 ± 10.8	NA	NA	27.0 ± 2.7	NA	NA	5.3 ± 3.5	FSL 1.5T	*p* <0.05 corrected	15
Weber et al. (5)	DM1 14 HC 20	37.2 ± 14.2 40.3 ± 12.5	NA 8/20	16.0 ± 9.6	574 ± 308	3.1 ± 0.8	28.2 ± 1.9	NA	Copy 33.3 ± 4.5 Recall 19.7 ± 9.0	10.1 ± 7.4	SPM2 1.5T	*p* <0.05 corrected	13
Serra et al. (8)	DM1 10 HC 16	41.8 ± 9.6 42.8 ± 12.9	4/6 7/9	NA	534.4 ± 539.3	2.7 ± 0.9	28.3 ± 2.1	A 48.8 ± 36.4 B 121.4 ± 100.5	NA	5.3 ± 3.5	SPM8 3T	*p* <0.05 corrected	15
Schneider-Gold et al. (11)	DM1 12 HC 33	45 ± 13 42 ± 14	4/8 19/14	18 ± 7	450(range 75–720)	3.3 ± 1.2	NA	NA	NA	7.6 ± 7.5	SPM8 3T	*p* <0.05 corrected	14
Minnerop et al. (6)	DM1 22 HC 22	42.1 ± 12.6 50.1 ± 9.0	13/9 11/22	13.2 ± 7.0	614 ± 306	3.6 ± 0.9	NA	A 33.45 ± 11.70 B 96.52 ± 48.75	NA	9.3 ± 7.8	SPM5 3T	*p* <0.05 corrected	13
Labayru et al. (10)	DM1 9 HC 12	30 ± 6.59 33.5 ± 8.32	5/4 7/5	NA	851.89 ± 444.77	2.56 ± 0.88	NA	NA	Copy 37.25 ± 10.38 Recall 41.38± 10.21	NA	FSL 1.5T	*p* <0.05 corrected	14
Labayru et al. (10)	DM1 12 HC 14	45.83 ± 9.05 43.64 ± 8.11	6/6 6/8	NA	362.83 ± 325.54	2.08 ± 1.0	NA	NA	Copy 33.3± 4.5 Recall 19.7± 9.0	NA	FSL 1.5T	*p* <0.05 corrected	15
Caso et al. (7)	DM1 51 HC 34	42 ± 10 45 ± 10	24/27 2,113	19.2 ± 8.5	750 ± 276	3.3 ± 0.7	NA	A 55.6 ± 21.6 B 156.6 ± 82.8	Copy 23.8 ± 5.5 Recall 13.1± 5.2	NA	SPM8 1.5T	*p* <0.05 corrected	15
Antonini et al. (4)	DM1 22 HC 22	33(the median), range 20–55 NA	9/13 NA	12.5(the median), range 1–35 NA	539 ± 365	NA	NA	NA	NA	NA	SPM99 1.5T	*p* <0.05 corrected	13

*DM1, myotonic dystrophy type 1; HC, healthy controls; NA, not applicable; F/, female/male; MIRS, muscular impairment rating scale; MMSE, mini mental state examination; TMT, trail-making test; ROCF, rey-osterrieth complex figure; BDI, Beck Depression Inventory score*.

### Regional Differences in GMV

**Figure 2:**
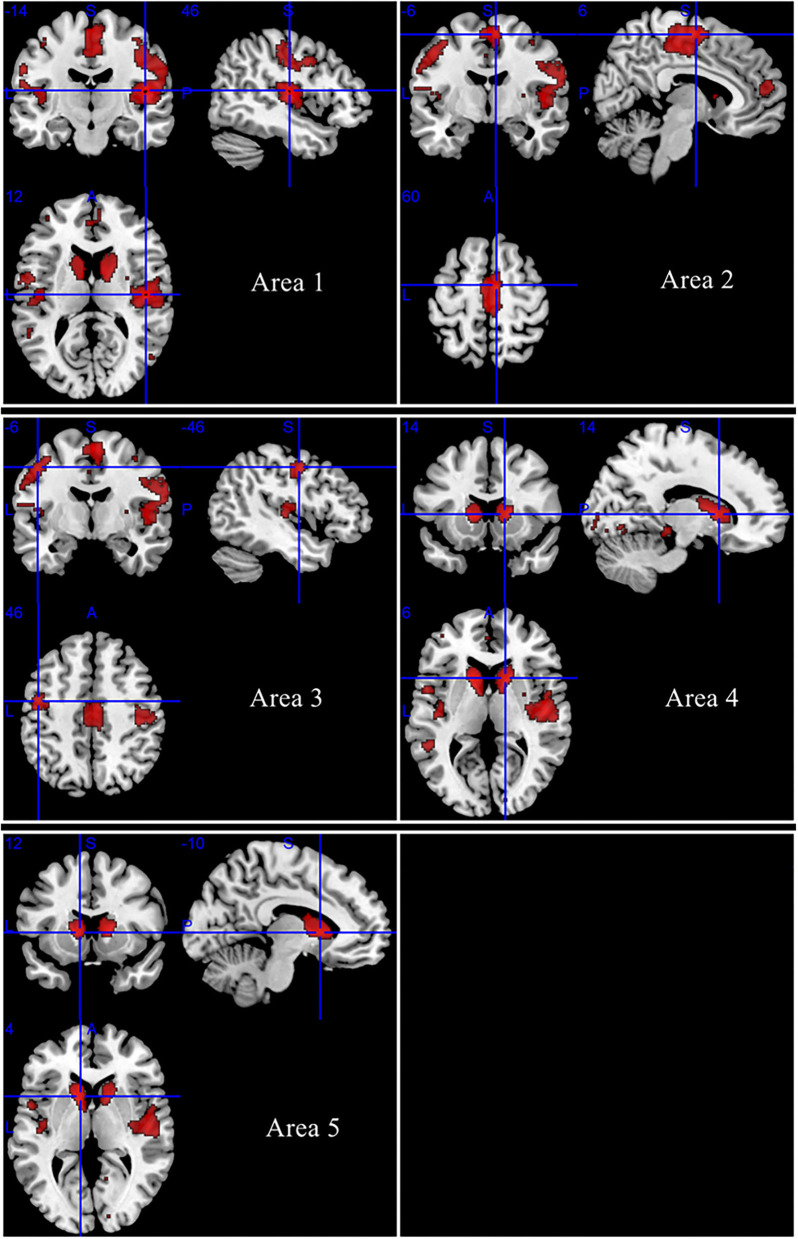
Regions of gray matter atrophy in patients with DM1 compared with HCs.

As shown in Figure 2, no increased GMV was detected in patients with DM1 compared with HCs. Reduction of GMV was found in bilateral rolandic operculum, bilateral posterior central gyrus, bilateral precentral gyrus, right insula, right heschl gyrus, right superior temporal gyrus, bilateral supplementary motor area, bilateral middle cingulate gyrus/paracingulate gyrus, left paracentral lobule, and bilateral caudate nucleus. The results of the meta-analysis are summarized in Table 2.

**Table 2:** The results of meta-analysis and jackknife sensitivity analysis.

**Region**	**Maximum**			**Egger test (*p*-value)**	**Clusters**		**Jackknife sensitivity analysis**
	**MNI coordinate (*x*, *y*, *z*)**	**SDM-Z**	***p*-Values**		**No. of voxels**	**breakdown**	
Area 1	46, −14, 12	−4.309	0.000008225	0.897	2,149	Right rolandic operculum, BA 48	9 out of 9
						Right postcentral gyrus, BA 43, BA4, BA3	9 out of 9
						Right insula, BA 48	9 out of 9
						Right precentral gyrus, BA 6, BA 4	9 out of 9
						Right heschl gyrus, BA 48	9 out of 9
						Right superior temporal gyrus, BA 48	9 out of 9
Area 2	6, −6, 60	−4.199	0.000013351	0.757	1,096	Right supplementary motor area, BA 6, BA 4	9 out of 9
						Left supplementary motor area, BA 6	9 out of 9
						Right median cingulate/paracingulate gyri	9 out of 9
						Left median cingulate/paracingulate gyri, BA 23	9 out of 9
						Left paracentral lobule, BA4	9 out of 9
Area 3	−46, −6, 46	−3.629	0.000142276	0.692	680	Left precentral gyrus, BA 6	9 out of 9
						Left rolandic operculum, BA 48	7 out of 9
						Left postcentral gyrus, BA 48, BA 4, BA 43	9 out of 9
Area 4	14, 14, 6	−4.065	0.000024021	0.701	414	Right caudate nucleus	9 out of 9
Area 5	−10, 12, 4	−4.168	0.000015318	0.992	379	Left caudate nucleus	9 out of 9

*BA, Brodmann area; MNI, montreal neurological institute*.

### Analyses of Sensitivity, Heterogeneity, and Publication Bias

A whole-brain jackknife sensitivity analysis showed that the GMV reductions in all regions derived from significant clusters were replicable in eight studies (Table 2). Heterogeneity analysis using *Q* and *I*^2^ (0.430%−5.359%) statistics showed no variability between studies. A quantitative assessment of the Egger's test (*p* = 0.337–0.992) showed no publication bias in all significant brain regions (Table 2), and funnel plots showed no significant asymmetry in all detected brain regions (Supplementary Figure 1).

### Subgroup Meta-Analyses

Considering the effects of different magnetic fields on gray matter, we divided nine studies into two parts, including six 1.5 T scanner studies and three 3 T scanner studies. A meta-analysis was conducted for a subgroup of 1.5 T scanners, we found that the results remained basically unchanged with only one additional significantly decreased cluster in the left middle temporal gyrus. Supplementary Table 2 details areas of gray matter atrophy in the 1.5 T scanner subgroup. However, only three 3 T scanner studies were unable to conduct a meta-analysis.

### Meta-Regression Analyses

Meta-regression analysis showed that longer disease duration was associated with severe atrophy in the left superior temporal gyrus, left caudate nucleus, and right rolandic operculum in patients with DM1. A lower Rey-Osterrieth Complex Figure (ROCF)-copy score was associated with severe atrophy of the left superior temporal gyrus and orbital part of the left middle frontal gyrus (see Table 3 for details). Age, onset age, MIRS score, GTC repeats, BDI score, MMSE score, and TMT score had no significant correlations with GMV changes.

**Table 3:** Meta-regression analyses of disease duration and ROCF-copy on GMV abnormal changes in DM1 patients.

**Region**	**Peak MNI coordinate (*x*, *y*, *z*)**	**SDM-Z**	***p*-Values**	**No. of voxels**
**Disease duration**				
Left superior temporal gyrus	−50, −8, 4	−3.694	0.00011057	402
Left caudate nucleus	−10, 12, 14	−3.311	0.0004651	98
Right rolandic operculum, BA 48	54, −10, 10	−3.313	0.00046164	69
**ROCF-copy**				
Left superior temporal gyrus, BA 48	−60, −14, 2	3.919	0.000044405	329
Left middle frontal gyrus, orbital part, BA 11	−26, 42, −20	3.308	0.00047064	91

*ROCF, rey-osterrieth complex figure; GMV, gray matter volume; DM1, myotonic dystrophy type 1; MNI, montreal neurological institute; BA, Brodmann area*.

### Family-Wise Error Correction

Family-wise error (FWE) was used to correct for thresholds with *p* < 0.05, and all results overlapped with uncorrected results.

## Discussion

This study is the first VBM-based quantitative meta-analysis of GMV changes in patients with DM1. Our study identified extensive GMV reduction in patients with DM1 both in cortical and subcortical structures, most of which are bilateral and symmetrical. Sensitivity analysis and FWE correction confirmed the robustness of the results.

In the current study, we detected that GMV reduction in cortex associated with motor areas in patients with DM1 through meta-analyses, such as bilateral rolandic operculum, precentral gyrus, postcentral gyrus, and supplementary motor area. Therefore, DM1 is not just a disease affecting the muscle in the traditional viewpoint, brain motor networks are also involved in (20). Rolandic operculum is located on the surface of the posterior central gyrus within the lateral fissure and is part of the language output network (21, 22). Lesions in this area may result in poor pronunciation or defective speech fluency (23, 24). Therefore, speaking and swallowing difficulties observed in patients with DM1 might partially ascribe to the impairment of the rolandic operculum besides the weakness and myotonia of the tongue and throat muscles. The precentral gyrus participates in the inhibition of involuntary movement and regulates movement through the basal ganglia (25). Electrophysiological experiment (26) and neuroimage-based meta-analysis studies found that motor areas (such as supplementary motor area, primary motor cortex, etc.) are involved in various stages of motor skill learning (27, 28). Pathological studies of DM1 showed neuron loss, development of abnormally neurofibrillary tangles (NFTs) or eosinophilic transparent inclusion bodies in extensive cortical regions, including the frontal lobe (29, 30). These findings suggest that the impairment of motor executive and planning function in DM1 is related to the central motor structure (31). An fMRI study found patients with DM1 had a larger cerebral blood-oxygen-level-dependent signal in the supplementary motor area during myotonia, suggesting that cortical function in high-order motor control areas is associated with the occurrence of myotonia (32), which also supported that the finding of GMV reduction in precentral gyrus identified in our study. The posterior central gyrus is one of the central areas of sensory input and utilization in the control of limb movement (33). A study using transcranial magnetic stimulation showed that the plasticity of the somatosensory cortex is involved in the consolidation of motor memory and motor learning (34, 35), and the earliest changes during learning are in the somatosensory cortex rather than the motor cortex (35). In a recent FDG-PET study, patients with DM1 showed the most significant abnormalities of glucose metabolism in bilateral frontal precentral and parietal postcentral regions (36), which also supported the finding of GMV reduction detected in our study.

In addition to identified atrophy areas associated with motor areas, our study also found GM atrophy in the superior temporal gyrus, heschl gyrus, cingulate gyrus, and insula, which may be associated with cognitive deficits and personality changes observed in patients with DM1, such as visual-spatial deficits, decreased attention and memory, and psychiatric symptoms like anxiety and depression (37). A recent study on cortical thickness found that there was a significant correlation between cortical thickness and the ability of patients with DM1 to correctly detect emotions. For example, there were significant associations between cortical thickness and sadness in the superior temporal gyrus, the right precentral gyrus, the right angular gyrus, and the left medial frontal gyrus bilaterally (38). An FDG-PET study found a good association between regions of hypoglucose metabolism and different neuropsychological impairments in patients with DM1. Frontal parietal dysfunctions were observed in the early stage of patients with DM1, however, memory and language impairments were observed in the late stage, suggesting the temporal lobe may be involved later (39, 40), which was also confirmed by the finding of neuronal loss, abnormal NFTs or eosinophilic hyaline inclusion bodies in the temporal lobe by postmortem studies (29, 30). The posterior cingulate gyrus plays a key role in the extraction of short-term memory (41, 42). Therefore, the GM atrophy of the cingulate gyrus identified in patients with DM1 may explain the poor short-term memory of patients with DM1. The insula involves different functions, such as the visceral sensory area, visceral motor area, motor association area, vestibular area, language area, and somatosensory area (43). A previous study found that patients with DM1 had a mildly decreased glucose metabolism in the insula (36). ROCF is a powerful and sensitive neuropsychological measure that reflects cognition in visual-spatial and memory functions. In our meta-regression analysis, the ROCF-recall score was positively correlated with GMV of the left superior temporal gyrus and middle frontal gyrus (orbital part). The middle frontal gyrus (orbital part) is a part of the prefrontal cortex that is closely related to thinking, memory of information, recall, and emotion. In addition, studies have found that the involvement of multimodal integrated network regions (such as insula, rolandic operculum, and anterior cingulated cortex) is also related to neuropsychological symptoms (44, 45). Therefore, the disconnection between primary sensory regions and higher cortical centers may be the basis of impaired higher cognitive function in patients with DM1 (10). A resting-state fMRI study also showed reduced amplitude of spontaneous activity in the middle frontal gyrus, cingulate gyrus, and supplementary motor area of patients with DM1 (46). These regions are components of the default mode network and executive control network, and play an important role in executive function and higher-order cognitive functions (47–49). In addition, a 4-year follow-up study found that the WM and GM of patients with DM1 were gradually affected, and the damage to working memory and visuospatial skills were significantly correlated with the changes in WM (50). This shows that the impairment of cognitive function is not only related to GM, WM also plays an important role.

In our meta-analysis, we found the subcortical regions with GM abnormalities mainly including the bilateral caudate nucleus, which is involved in the regulation of muscle tone, postural reflex, and complex behavior (51). Postmortem study on patients with DM1 also revealed neuronal loss, abnormal NFTs, or eosinophilic transparent inclusions in the basal ganglia (29, 30). Combined with our findings, basal ganglia may be partly responsible for myotonia bedsides repeated depolarization of the muscle membrane, and fixed thinking in patients with DM1 (52).

In the meta-regression analysis, we found that disease duration was correlated with GM abnormalities in the left superior temporal gyrus, left caudate nucleus, and right rolandic operculum, suggesting that the impairment of the central nervous system was increased with the progression of the disease, which was consistent with a previous five-year follow-up study (53). A recent 9-year follow-up study found that cognitive score progression was related to disease duration (54).

Our study has the following limitation: First, VBM processing methods and magnetic field intensity of MRI scanning among different studies are different, which cannot completely exclude the differences in the measurement process. Second, some included studies had incomplete information and used varied evaluation scales and criteria. Third, we did not analyze the abnormalities of white matter because of the lack of sufficient literature. Finally, this study only studied the GMV changes of patients with DM1 at one time point, and the change of its volume over time was not explored.

## Conclusion

The current voxel meta-analysis found patients with DM1 have extensive cortical and subcortical gray matter atrophy, including rolandic operculum, posterior central gyrus, precentral gyrus, supplementary motor area, insula, heschl gyrus, superior temporal gyrus, middle cingulate gyrus/paracingulate gyrus, and caudate nucleus. Regional GM abnormalities were associated with disease duration and ROCF-recall. Our findings provide evidence of central neural anatomic changes for motor dysfunction and neuropsychiatric symptoms in patients with DM1. Future studies on longitudinal brain structure alteration are needed.

## Data Availability Statement

The original contributions presented in the study are included in the article/Supplementary Material, further inquiries can be directed to the corresponding author.

## Ethics Statement

Written informed consent was obtained from the individual(s), and minor(s)' legal guardian/next of kin, for the publication of any potentially identifiable images or data included in this article.

## Author Contributions

QJ contributed with conception and execution, data collection, statistical analysis, and writing the manuscript. JL contributed to the conception, data collection, and review and editing of the manuscript. CL and YH contributed to the execution and data collection. HS contributed with conception and organization, manuscript review and critique, and was responsible for overall content as the guarantor. All authors contributed to the article and approved the submitted version.

## Funding

This article was supported by the 1.3.5 project for disciplines of excellence, West China Hospital, Sichuan University (No. ZYJC18038) and the Science Foundation of Chengdu Science and Technology Bureau (Grant No. 2019-YF05-00307-SN).

## Conflict of Interest

The authors declare that the research was conducted in the absence of any commercial or financial relationships that could be construed as a potential conflict of interest.

## Publisher's Note

All claims expressed in this article are solely those of the authors and do not necessarily represent those of their affiliated organizations, or those of the publisher, the editors and the reviewers. Any product that may be evaluated in this article, or claim that may be made by its manufacturer, is not guaranteed or endorsed by the publisher.
