# Supplementary material for: Gray Matter Abnormalities in Myotonic Dystrophy Type 1: A Voxel-Wise Meta-Analysis
Source: Front Neurol. 2022 Jul 7;13:891789. doi: 10.3389/fneur.2022.891789 (PMC9301187; doi:10.3389/fneur.2022.891789)

Supplementary Table 1

| **Supplementary Table 1. Criteria for quality assessment of individual studies** |
| --- |
| \| **Category 1: Sample characteristics (10)** \| \| --- \| \| 1. Patients were evaluated with specific standardized diagnostic criteria (1) \| \| 1. Important demographic data (age and gender) were reported with mean (or median) and standard deviations (or range)) (2) \| \| 1. Healthy comparison subjects were evaluated to exclude psychiatric and medical illnesses and demographic data was reported (1) \| \| 1. Important clinical variables (e.g. illness duration, TRS scores) were reported with mean (or median) and standard deviations (or range)) (2) \| \| 1. Sample size per group ≧10 (1) \| \| **Category 2: Methodology and reporting (10)** \| \| 1. All neuroanatomic measurements were taken without considering group assignment or subject identity (1) \| \| 1. Magnet strength at least 1.5T (1) \| \| 1. MRI slice-thickness ≤ 3 mm and more than 1 slice was identified and traced (1) \| \| 1. The acquisition and preprocessing techniques were clearly described so that they could be reproduced (1) \| \| 1. Measurements were clearly described so that they could be reproduced (1) \| \| 1. Coordinates were reported in a standard space unless there was no significant difference (1) \| \| 1. Significant results are reported after correction for multiple testing using a standard statistical procedure (FDR, FWE or permutation-based methods) (1) \| \| 1. Conclusions were consistent with the results obtained and the limitations were discussed (1) \| \| **Total**  /15 \| |

Supplementary Table 2 Regional differences in gray matter volumes in a subgroup meta-analyses of studies with 1.5T MRI

| Region | Maximum | | | | Clusters | |  |
| --- | --- | --- | --- | --- | --- | --- | --- |
|  | **MNI coordinate (x,y,z)** | **SDM-Z** | **P-values** | **No. of voxels** | | **Breakdown** | |
| Area 1 | 62,-12,28 | -3.731 | 0.000095189 | 653 | | Right postcentral gyrus, BA 43,BA 4 | |
|  |  |  |  |  | | Right rolandic operculum, BA 48 | |
| Area 2 | 4,-22,52 | -3.704 | 0.000105917 | 584 | | Right supplementary motor area, BA 6,BA 4 | |
|  |  |  |  |  | | Left supplementary motor area, BA 6 | |
|  |  |  |  |  | | Right median cingulate/paracingulate gyri | |
| Area 3 | -12, 12, 6 | -3.799 | 0.000072777 | 262 | | Left caudate nucleus | |
| Area 4 | -58,-12,32 | -3.397 | 0.000340879 | 163 | | Left postcentral gyrus, BA 43 | |
| Area 5 | 10,4,14 | -3.540 | 0.000200093 | 135 | | Right caudate nucleus | |
| Area 6 | 18,-28,-6 | -3.543 | 0.000198007 | 106 | | Right median network, cingulum | |
| Area 7 | -50,-44,8 | -3.343 | 0.000414133 | 98 | | Left middle temporal gyrus, BA 22,BA21 | |

Supplementary Figure 1 Results of funnel plot analysis


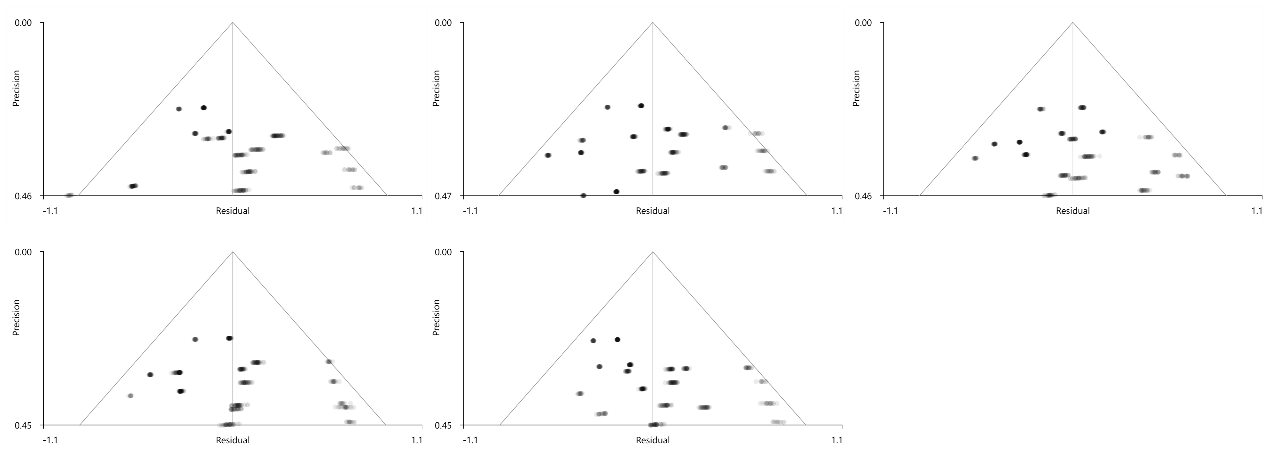

Supplement: Supplementary file 1 [file Data_Sheet_1.docx]
